# Widely Invasive Follicular Thyroid Carcinoma With Extensive Nodal Metastases and Subsequent Atypical Locoregional Recurrences: A Case Report

**DOI:** 10.7759/cureus.110971

**Published:** 2026-06-16

**Authors:** Lucas E Cohen, Milo J Cohen, Alena I Padilla Rocca, Shawhin Karimi

**Affiliations:** 1 General Medicine, The Kinkaid School, Houston, USA; 2 General Medicine, College of Natural and Health Sciences, University of Tampa, Houston, USA; 3 Endocrinology, Houston Endocrinology Center, Houston, USA

**Keywords:** follicular thyroid carcinoma, locoregional recurrence, lymph node metastasis, substernal goiter, widely invasive

## Abstract

Follicular thyroid carcinoma (FTC) is a well-recognized thyroid malignancy classically known for hematogenous spread, making extensive lymph node involvement at diagnosis an atypical finding. Here, we present the case of a 49-year-old man with widely invasive FTC presenting with an extraordinary burden of metastatic cervical lymph nodes at diagnosis. He subsequently developed two sequential locoregional recurrences at atypical locations, the retropharyngeal space and the superior mediastinum, and ultimately achieved no evidence of disease. This case highlights the potential for widely invasive FTC to present with an extraordinary nodal burden and to recur at unusual anatomic locations not typically associated with FTC. It also demonstrates that meaningful disease control was achieved in this case despite aggressive disease features, provided recurrences are detected early and managed promptly with a multidisciplinary approach. Clinicians should maintain a low threshold for biopsy of new findings on surveillance imaging in patients with a history of widely invasive FTC.

## Introduction

Follicular thyroid carcinoma (FTC) is the second most common type of thyroid cancer, comprising approximately 10% of all cases [1]. Most thyroid cancers spread through the lymphatics, but FTC is different; it typically spreads through the bloodstream, commonly reaching the lungs and bones. As a result, lymph node involvement at the time of diagnosis is relatively uncommon. Studies have reported nodal metastases in only 2.0% of minimally invasive FTC cases and 9.8% of widely invasive cases [2]. Spread to lymph nodes outside the central neck is even less common and not well described in the literature.

Cases of FTC with large numbers of involved lymph nodes, particularly without simultaneous spread to distant organs, have only rarely been reported [3]. When a thyroid tumor also extends into the chest (substernal extension), surgical management becomes significantly more complex. The combination of substernal disease and extensive lymph node involvement is exceptionally uncommon.

When FTC comes back after initial treatment, it can be difficult to manage, especially when it returns in locations that are less easily accessible for surgery. Recurrence in the retropharyngeal space or the superior mediastinum is rare, and the two occurring one after another in the same patient has not been previously described.

Herein, we report a case of a 49-year-old man with widely invasive FTC who presented with a massively enlarged thyroid extending into the superior mediastinum and 43 metastatic lymph nodes. He subsequently developed two sequential recurrences at unusual anatomic sites, the retropharyngeal space and the superior mediastinum. This case illustrates how widely invasive FTC can behave unexpectedly and highlights the need for close, long-term follow-up in patients with extensive disease at presentation.

## Case presentation

A 49-year-old man presented to his endocrinologist for clinical evaluation after his wife noticed a lump in his neck. Initial laboratory workup revealed a thyroid-stimulating hormone (TSH) of 2.6 mIU/L. Thyroid ultrasound demonstrated multiple nodules suggestive of a multinodular goiter, and the largest measured 6.0, 4.3, 3.2, 3.0, and 1.9 cm (Figure 1). Two weeks after presentation, fine-needle aspiration (FNA) of two nodules in the left lobe and isthmus returned results of atypia of undetermined significance (Bethesda Category III).

**Figure 1:**
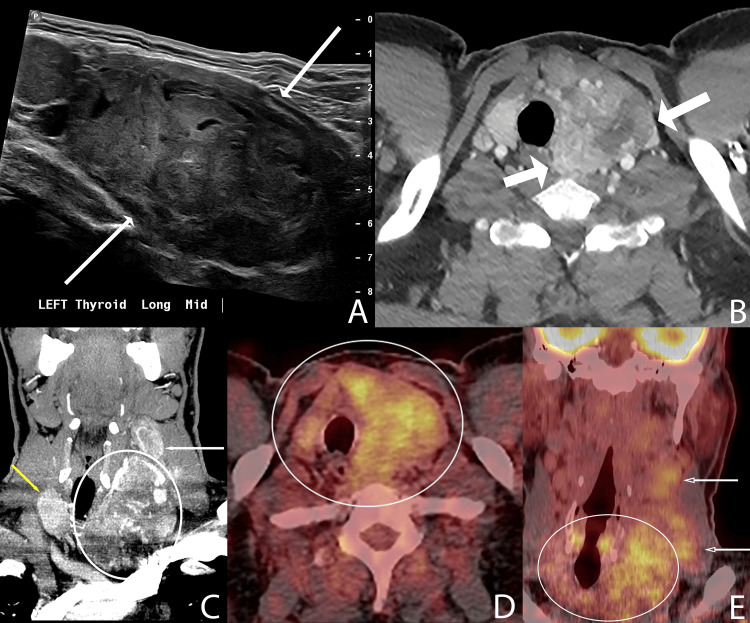
Preoperative imaging at initial presentation. (A) Longitudinal ultrasound image of the left lobe of the thyroid shows massive heterogeneous enlargement of the left lobe of the thyroid with multiple nodular areas, suggestive of multinodular goiter (white arrows). Ultrasound-guided fine-needle aspiration was recommended. (B) Axial postcontrast CT image 2.5 months after initial presentation again shows massive enlargement of the left lobe of the thyroid with very heterogeneous enhancement (white arrows) and tracheal deviation, highly concerning for malignancy. (C) Coronal postcontrast CT thick-slice reconstruction image (same CT examination) redemonstrates the abnormal left lobe of the thyroid (open circle). Also identified on this image is the normal appearance of the right lobe of the thyroid (yellow arrow) and an abnormal level 3 left cervical lymph node (white arrow). (D) Axial FDG PET/CT image reveals abnormal increased metabolic activity/FDG uptake throughout the enlarged left lobe of the thyroid. (E) Coronal FDG PET/CT image redemonstrates the same abnormality in the left lobe of the thyroid (open cervical) with abnormal increased metabolic activity/FDG uptake throughout the level 3 and level 4 left cervical lymph nodes (white arrows). FDG: fluorodeoxyglucose

Given the large nodule burden and indeterminate biopsy results, cross-sectional imaging was obtained. A CT of the neck approximately 2.5 months after presentation (Figure 1) showed massive enlargement of the left thyroid lobe with extension into the superior mediastinum, rightward deviation of the trachea and larynx, and extensive ipsilateral cervical lymphadenopathy. All findings were fluorodeoxyglucose (FDG)-avid at PET/CT (Figure 1). FNA and core biopsy of a left cervical lymph node were performed two weeks later and confirmed metastatic FTC, which established the diagnosis. At that time, serum thyroglobulin (TG) was 2,840.60 ng/mL (normal range, 1.59-50.03 ng/mL) and serum anti-TG was <0.9 IU/mL (normal range, 3.9 IU/mL).

Approximately four months after initial presentation, the patient underwent total thyroidectomy and left level 2-6 neck dissection through a standard cervical incision, without the need for sternotomy or thoracotomy. This interval reflects the logistical complexity of coordinating care at a tertiary referral center for a patient residing at a considerable distance, as well as the time required to complete the necessary preoperative workup, including cross-sectional imaging, metabolic imaging, and tissue confirmation via lymph node biopsy. Final surgical pathology showed widely invasive FTC with extrathyroidal extension, extensive angioinvasion, and tumor thrombus within an unnamed ipsilateral superficial vein. A total of 43 lymph nodes were positive for metastatic disease.

Approximately seven months after presentation, the patient received 177.8 mCi of I-131 radioactive iodine therapy. At the time of treatment, stimulated TG was 129.66 ng/mL and serum anti-TG was 2.9 IU/mL. The post-treatment scan showed uptake in the right thyroid bed, left submandibular lymph nodes, and right neck/supraclavicular region, with no evidence of distant metastases (images unavailable).

Approximately 13 months after presentation, surveillance CT of the neck identified a new abnormal retropharyngeal lymph node, representing the first locoregional recurrence (Figure 2). Three months later, the patient underwent transoral robotic surgery (TORS) with successful excision of the retropharyngeal node.

**Figure 2:**
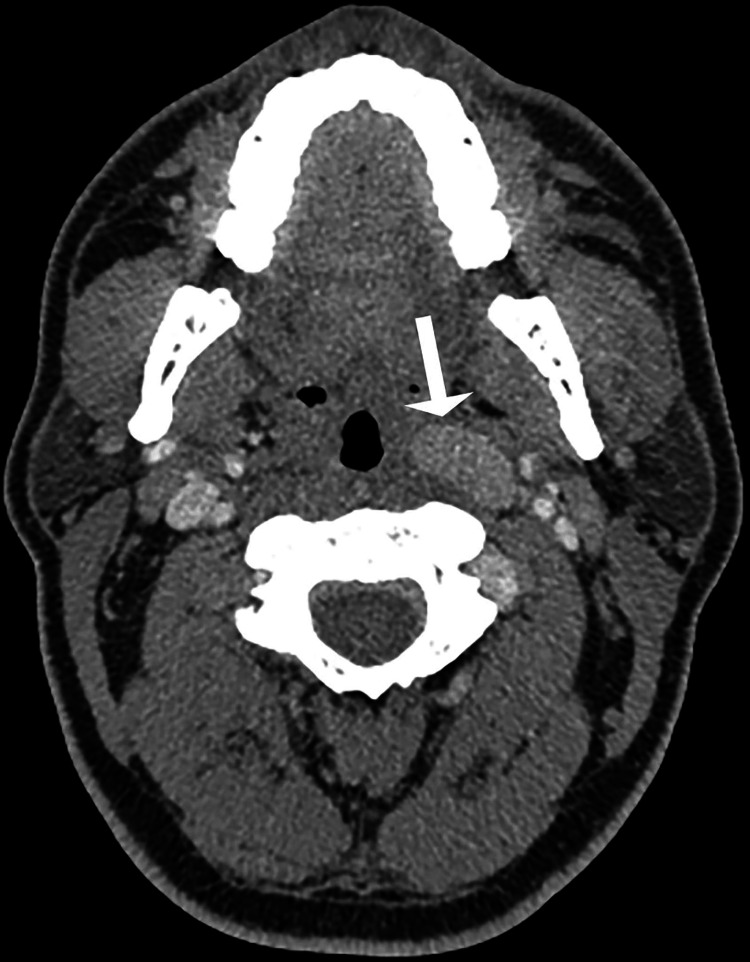
Postoperative imaging reveals the patient’s first nodal recurrence. An axial CT image 8 months after thyroidectomy and lymph node dissection at the level of the oropharynx shows an abnormally enlarged left level 2 cervical node (retropharyngeal, white arrow). This was resected during transoral robotic surgery 3 months later.

Approximately 19 months after presentation, surveillance CT revealed a new 2.3 cm nodule in the low right thyroidectomy bed (Figure 3). FDG PET/CT and ultrasound-guided FNA (Figure 3) one month later confirmed an FDG-avid follicular neoplasm consistent with the patient's known follicular carcinoma, representing the patient’s second locoregional recurrence. Three months after this latest recurrence, the patient underwent revision level 6 and 7 dissection, and the patient has had no evidence of disease at approximately two months following this procedure.

**Figure 3:**
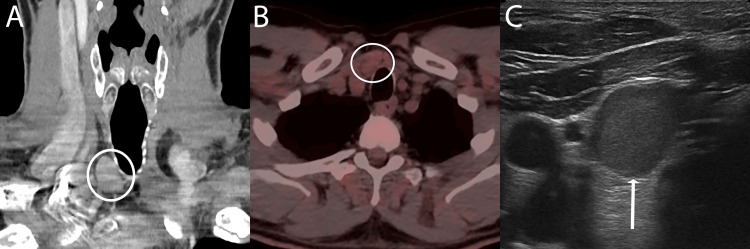
Surveillance imaging after transoral robotic surgery led to the second diagnosis of recurrent follicular neoplasm. (A) Coronal CT image of the neck almost 3 months after resection of the retropharyngeal node, approximately 15 months after initial thyroidectomy and lymph node dissection, reveals a new mass (open circle) at the base of the neck in the prior location of the right lobe of the thyroid. (B) Subsequently performed axial FDG PET/CT image shows mild FDG uptake within this mass (open circle), concerning for recurrent disease. (C) Transverse ultrasound image of the thyroid bed redemonstrates this mass (white arrow). An ultrasound-guided fine-needle aspiration performed on the same day was positive for follicular neoplasm. FDG: fluorodeoxyglucose

## Discussion

This case is unusual in several important ways. FTC is known for spreading hematogenously rather than through the lymphatics, yet this patient presented with 43 metastatic nodes, a burden that far exceeds what is commonly described in the FTC literature. He also had a massively enlarged thyroid, which extended into the superior mediastinum, two sequential recurrences at atypical anatomic locations, and ultimately achieved no evidence of disease after aggressive multimodal management.

Cervical lymph node metastases are seen in only 2.0% of minimally invasive FTC cases and 9.8% of widely invasive cases at diagnosis [2]. The 43 positive nodes in this patient, therefore, represent an exceptionally high nodal burden for this cancer type. To our knowledge, the largest previously reported case of FTC with cervical lymph node involvement without distant metastases involved only nine positive nodes [3], compared to the 43 in the present case. This suggests that the nodal burden described herein may be the highest documented for FTC in the literature. Table 1 compares the present case with previously published cases of FTC demonstrating either substantial nodal involvement or atypical recurrence patterns, further highlighting the exceptional nodal burden and unusual sequential recurrences observed here.

**Table 1:** Comparison of selected published cases of follicular thyroid carcinoma with atypical nodal or recurrence patterns. FTC: follicular thyroid carcinoma; NED: no evidence of disease; NR: not reported; RAI: radioactive iodine; TKI: tyrosine kinase inhibitor

Feature	Present case	Ye et al., 2022 [3]	Lee and Park, 2020 [4]	Kim et al., 2021 [5]	Staubitz and Musholt, 2020 (review article) [6]
Age/sex	49/M	66/M	81/F	51/F	50/M
Histology	Widely invasive FTC	Angioinvasive FTC	Minimally invasive FTC	Widely invasive FTC	FTC (capsular and vascular invasion)
Tumor size	8.2 cm	16.0 cm	7.5 cm	8.9 cm	6.0 cm
Substernal extension	Yes	Partial (retrosternal)	No	No	No
Metastatic nodes at presentation	43	9	0	0	5 of 42
Distant metastases	No	No	Yes (lung, 5 months post-op)	Yes (lung, hilar, 5.5 years post-op)	Yes (lung, bone, after recurrence)
Atypical recurrence sites	Yes (retropharyngeal space, superior mediastinum)	No (cervical only)	Yes (mediastinal)	Yes (axillary)	No (cervical only)
Outcome	NED at 2 months	Awaiting RAI at time of writing	Died at 15 months	Died at 7 years	Ongoing TKI therapy

Substernal extension of the primary tumor made surgical planning more complex. Most substernal goiters can be removed safely through a cervical incision without opening the chest; in a series of 212 substernal goiter procedures, only two cases required sternotomy [7]. That was the approach taken here, and the fact that the surgical team was able to fully address both the primary tumor and the extensive nodal disease through a cervical approach alone reflects careful preoperative planning and surgical expertise.

The pathologic findings further underscore the aggressive nature of this case. In one large series, widely invasive FTC was associated with a recurrence rate of 38% and an overall disease-related mortality of 62% after a mean follow-up of 7.5 years [8]. It has also been identified as an independent predictor of worse cancer-specific survival (HR 3.6) and shorter disease-free interval (HR 2.5) [9]. The extensive vascular invasion and tumor thrombus seen on pathology in this case are consistent with the aggressive biology of this subtype and may partly explain the atypical patterns of spread observed.

The first recurrence in the retropharyngeal space is particularly unusual. Retropharyngeal lymph node metastases are rarely encountered in thyroid cancer, and their occurrence in FTC specifically is not well described. The use of TORS for excision in this location was an appropriate and minimally invasive approach that allowed for complete resection without significant morbidity.

The second recurrence in the level VI/VII region, the low thyroidectomy bed and superior mediastinum, may reflect either true de novo spread or persistent microscopic disease in a territory that is difficult to fully clear surgically at the time of initial resection. For cases of mediastinal recurrence not amenable to surgery, external beam radiotherapy (EBRT) at a median dose of approximately 6,300 cGy has been shown to achieve two-year locoregional control rates of 86% in advanced or recurrent non-anaplastic thyroid cancer [10]. In this patient, surgical resection remained feasible and was performed.

Distant metastases at diagnosis have been identified as the strongest independent prognostic factor for cancer-specific survival in widely invasive FTC [11]. This is notable in the context of the present case, where the patient had an extraordinary nodal burden but no distant metastases at any point in his disease course. His pattern of recurrence was driven completely by locoregional disease, which may in part explain why meaningful disease control was ultimately achievable. This raises the question of whether patients with high nodal burden but no distant metastases represent a distinct clinical subset of widely invasive FTC with a more favorable trajectory than the overall survival data would suggest.

The extent of vascular invasion has also been shown to independently predict distant metastasis-free survival in widely invasive FTC, with patients having two or more foci of vascular invasion showing a 10-year distant metastasis-free survival rate of only 56.8% compared to 96.8% in those with fewer foci [12]. The extensive vascular invasion and tumor thrombus present in this case would place him in a high-risk category by this metric, making his no-evidence-of-disease status after multimodal management particularly noteworthy.

Ultimately, this patient achieved no evidence of disease following two reoperations for recurrent disease, which is an encouraging outcome given the aggressive features at presentation. It should be noted that this reflects only approximately two months of follow-up at the time of this writing, and long-term outcomes remain uncertain. Nevertheless, his case illustrates that meaningful disease control is possible in widely invasive FTC even with extensive initial disease, provided that recurrences are detected early and managed promptly with a multidisciplinary approach.

## Conclusions

We present a rare case of widely invasive FTC with substernal extension and 43 positive lymph nodes at initial diagnosis, followed by two sequential recurrences at anatomically atypical sites, the retropharyngeal space and the superior mediastinum. Widely invasive FTC is an aggressive disease associated with high rates of recurrence and disease-related mortality, and this case is a clear example of that potential. Despite these aggressive features, this patient achieved no evidence of disease after multimodal management including total thyroidectomy, neck dissection, radioactive iodine therapy, TORS, and revision neck dissection. This early outcome highlights the importance of early detection of recurrence and prompt, aggressive management, even at unusual anatomic sites. While encouraging, it must be acknowledged that the patient has been free of disease for only approximately two months at the time of this writing, and the durability of this response remains to be seen given the aggressive nature of the disease and the patient's history of two prior recurrences within two years. The absence of distant metastases throughout his disease course may have contributed to his favorable outcome despite the extent of locoregional disease.

Clinicians should be aware that recurrence in widely invasive FTC can occur at unusual locations, including the retropharyngeal space and the superior mediastinum, and should maintain a low threshold for biopsy of new findings on surveillance imaging. A multidisciplinary approach involving endocrinology, surgery, nuclear medicine, and radiology is critical to optimizing outcomes in complex cases such as this one.
